# Effects of naltrexone are influenced by childhood adversity during negative emotional processing in addiction recovery

**DOI:** 10.1038/tp.2017.34

**Published:** 2017-03-07

**Authors:** G Savulich, R Riccelli, L Passamonti, M Correia, J F W Deakin, R Elliott, R S A Flechais, A R Lingford-Hughes, J McGonigle, A Murphy, D J Nutt, C Orban, L M Paterson, L J Reed, D G Smith, J Suckling, R Tait, E M Taylor, B J Sahakian, T W Robbins, K D Ersche, ICCAM Platform

**Affiliations:** 1 Department of Psychiatry, University of Cambridge, Cambridge, UK; 2 Behavioural and Clinical Neuroscience Institute, University of Cambridge, Cambridge, UK; 3 Department of Medical and Surgical Sciences, University Magna Graecia, Catanzaro, Italy; 4 Department of Clinical Neurosciences, University of Cambridge, Cambridge, UK; 5 Cognition and Brain Sciences Unit, Medical Research Council, Cambridge, UK; 6 Institute of Brain, Behaviour and Mental Health, University of Manchester, Manchester, UK; 7 Centre for Neuropsychopharmacology, Imperial College London, London, UK; 8 Department of Psychology, University of Cambridge, Cambridge, UK

## Abstract

Naltrexone is an opioid receptor antagonist used in the management of alcohol dependence. Although the endogenous opioid system has been implicated in emotion regulation, the effects of mu-opioid receptor blockade on brain systems underlying negative emotional processing are not clear in addiction. Individuals meeting criteria for alcohol dependence alone (*n*=18, alcohol) and in combination with cocaine and/or opioid dependence (*n*=21, alcohol/drugs) and healthy individuals without a history of alcohol or drug dependence (*n*=21) were recruited. Participants were alcohol and drug abstinent before entered into this double-blind, placebo-controlled, randomized, crossover study. Functional magnetic resonance imaging was used to investigate brain response while viewing aversive and neutral images relative to baseline on 50 mg of naltrexone and placebo. We found that naltrexone modulated task-related activation in the medial prefrontal cortex and functional connectivity between the anterior cingulate cortex and the hippocampus as a function of childhood adversity (for aversive versus neutral images) in all groups. Furthermore, there was a group-by-treatment-by-condition interaction in the right amygdala, which was mainly driven by a normalization of response for aversive relative to neutral images under naltrexone in the alcohol/drugs group. We conclude that early childhood adversity is one environmental factor that influences pharmacological response to naltrexone. Pharmacotherapy with naltrexone may also have some ameliorative effects on negative emotional processing in combined alcohol and drug dependence, possibly due to alterations in endogenous opioid transmission or the kappa-opioid receptor antagonist actions of naltrexone.

## Introduction

Emotions have a critical role in the development, maintenance and successful treatment of addiction.^1, 2, 3^ Suppression of negative affective states such as anxiety and withdrawal symptoms is one motivational pathway to support the consumption of alcohol.^4, 5^ As negatively reinforced drinking becomes more pronounced, negative affective states increase, thereby escalating alcohol intake and raising vulnerability to relapse after treatment.^6, 7^ Negative reinforcement is driven by activation of stress-induced neurocircuitry in what is widely referred to as the ‘dark side' view of addiction.^8, 9^ Negative emotion is thus a key affective process that could be targeted by treatment interventions for addiction.

Functional abnormalities during negative emotional processing have been found in limbic and cortical networks in substance dependence.^1^ Threat-related reactivity of the amygdala, for example, is strongly associated with negatively reinforced problem drinking.^10^ Connectivity between the amygdala and the hippocampus is associated with maladaptive emotional processing,^11^ and alterations in hippocampal network activation and connectivity have been shown to predict relapse.^12^ The prefrontal cortex has extensive connections with subcortical structures that regulate emotional processing, including the amygdala.^13^ Alcohol and drug exposure impairs emotion regulation in this region, with interconnected medial and cingulate networks showing enhanced reactivity to arousing stimuli and reduced capacity to suppress negative affect.^14^ The medial prefrontal cortex (mPFC) and anterior cingulate cortex (ACC) also act to appraise and regulate negative emotions.^15^ These cortical areas over-activate in response to substance-related and naturally evocative stimuli^16^ and, together with limbic regions associated with impaired emotional processing in addiction, are candidate loci for pharmacological intervention.

Naltrexone is one pharmacotherapy used in the management of alcohol dependence that works by modulating opioid control of dopaminergic cell firing in the ventral tegmental area, thus preventing an increase in dopaminergic activity.^17^ The endogenous opioid system has been implicated in emotion regulation.^18^ There is some evidence of naltrexone dampening responses to negative emotional stimuli in healthy adults,^19^ although this likely reflects stress-reducing effects of the potent kappa-opioid receptor (KOR) antagonist actions of naltrexone. mu-opioid receptor (MOR) antagonism is known to precipitate withdrawal symptoms in humans with current^20^ and previous^21^ opiate use. MOR antagonism also precipitates aversive consequences of withdrawal from chronic opioid exposure in animals.^22, 23^ Naltrexone increases negative emotions in response to stress- and drug-related images in individuals with opioid dependence^24^ and increases anxiety in response to drug-related films in individuals with alcohol and cocaine dependence.^25^ However, the efficacy of naltrexone treatment has been shown to be associated with greater naltrexone-induced aversion^26^ (that is, the more negative the aversive stimulus, the greater the treatment response). This may be due, in part, by activation of the hypothalamic–pituitary–adrenal (HPA) axis associated with all substances of abuse potential. Specifically, naltrexone-induced adrenocorticotropic hormone and cortisol levels are thought to reduce craving in individuals with alcohol dependence.^27^ Exposure to psychological trauma, another form of aversion, is also known to contribute to individual treatment responses to naltrexone.^28^ This is consistent with preclinical evidence showing that early environmental adversity is associated with better treatment effects of naltrexone,^29^ suggesting that adversity experienced in early stages of development upregulates endogenous opioid function.

Childhood adversity has long been known to be common in substance use disorders, with at least two-thirds of alcohol- or drug-dependent adults reporting a history of physical, sexual or emotional abuse.^30^ Childhood adversity leads to more illicit substance use and increases the risk of dependence in adulthood.^31, 32, 33^ Combined alcohol and drug dependence is particularly harmful, as individuals dependent on both consume more units of alcohol and have greater incidence and severity of psychiatric illness than individuals dependent on alcohol alone.^34^ The heterogeneity of alcohol use disorders, including concurrent non-alcohol drug use, necessitates development of more tailored treatment approaches.^35, 36^ However, patients are typically categorized according to their primary dependency or by the drug for which they seek treatment.^37^ Personalized assessment and specialized treatments addressing the effects of combined alcohol and drug-taking behavior are often omitted, thus reducing the potential for more successful recovery.^6^

We used functional magnetic resonance imaging (fMRI) to investigate brain response to aversive and neutral images. We sought to determine the effects of naltrexone at standard dose (50 mg) during negative emotional processing between groups dependent on alcohol alone, dependent on alcohol and drugs (both in abstinence) and healthy control volunteers. On the basis of previous research showing altered activation in limbic and cortical networks during negative emotional processing in substance-dependent individuals,^1, 10, 11, 12, 13, 14, 15^ including the processing of evocative^16^ and negative emotional images,^38, 39^ we hypothesized that the dependent groups would show increased activation in the amygdala, the mPFC and the hippocampus in response to aversive images. In light of preclinical evidence,^29^ we further hypothesized that these effects would be modulated by naltrexone depending on the degree of childhood adversity experienced.

## Materials and methods

### Participants

This was a double-blind, placebo-controlled, randomized, crossover study involving three sites (Imperial College London, University of Cambridge, University of Manchester; ICCAM). Full details of the ICCAM platform are reported elsewhere.^40, 41, 42^ Briefly, inclusion criteria were fluency in English; age 20–64; meeting DSM-IV criteria^43^ for alcohol, cocaine, amphetamine or opiate dependence; and abstinence from alcohol or drugs for at least 4 weeks prior to the experimental medicine sessions. Control participants did not meet DSM-IV criteria for any disorder. Exclusion criteria for all participants were use of medication that could not be paused for the study duration; current primary Axis I or neurological diagnosis; current or past psychiatric history (excluding lifetime or secondary history of anxiety or depression); and MRI contraindications. All participants screened negative (using urine samples) for amphetamines, barbiturates, cocaine, opiates, cannabinoids and benzodiazepines.

Here, we compared the effects of naltrexone between individuals who were only dependent on alcohol from individuals who in addition to alcohol were also dependent on cocaine and/or opiates. This distinction was made due to evidence showing that individuals dependent on alcohol and drugs have more severe psychopathology,^34^ and differences in brain function^44^ and structure^45^ compared with individuals dependent on alcohol alone. Control participants with a history of heavy alcohol or drug use were also excluded due to known associated functional and structural abnormalities.^46^ Heavy alcohol use was defined as a score higher than eight on the Alcohol Use Disorders Identification Test (AUDIT).^47^ Heavy drug use was defined by clinicians of the ICCAM team using the following criteria: more than 300 pills (lifetime) for MDMA; daily/almost daily THC use for more than one year and more than 2 spliffs; and more than once a week for more than six months and more than 1 g for amphetamines. On the basis of these criteria, patients with drug dependence but without alcohol dependence (*n*=11) and control participants with a history of heavy alcohol or drug use (*n*=9) were excluded.

The final alcohol group (alcohol) comprised 18 individuals meeting criteria for alcohol dependence only. The alcohol and drugs group (alcohol/drugs) comprised 21 individuals meeting criteria for alcohol dependence and the following drug dependencies: cocaine (42.9%); cocaine and opiates (47.6%); amphetamines (4.8%); and opiates (4.8%). The control group comprised 21 individuals without a history of alcohol or drug dependence.

### Baseline assessment

All participants completed the Wechsler Test of Adult Reading (WTAR)^48^ to measure verbal intelligence and control volunteers also completed the AUDIT to screen for harmful drinking patterns. To investigate the effects of individual variations of perceived stress and adverse childhood experiences on emotional processing, participants completed the Perceived Stress Scale (PSS-14)^49^ and the Childhood Trauma Questionnaire (CTQ).^50^ For the PSS-14, we used the total score and for the CTQ we calculated an abuse composite score from three abuse subscales, that is, physical, sexual and emotional abuse.^51^ In light of prior research suggesting the effects of naltrexone might be modulated by variations in locus of control beliefs,^52^ participants completed Rotter's locus of control scale^53^ and a drug-related version.^51^

### Procedure

This study received ethical approval from the West London and Gene Therapy Advisory Committee National Research Ethics Service committee (11/H0707/9). Participants provided consent, basic demographic information and baseline assessment measures. For the experimental medicine sessions, participants completed a urine screen and alcohol breath and pregnancy tests. Participants screening negative were dosed 2 h prior to the MRI scan. The experimental medicine sessions consisted of administering placebo or 50 mg of naltrexone in a counterbalanced order. The Beck Depression Inventory (BDI-II)^54^ and the Spielberger-State Anxiety Inventory (STAI)^55^ were administered to evaluate current mood on the day of testing. Smoking was permitted up to 1 h before scanning; caffeine was only permitted in the morning. Participants were given a snack on arrival but subsequent food intake was restricted to ensure full drug absorption. The experimental medicine sessions were separated by at least 1 week.

### Evocative images task

The evocative images task probes negative arousal to aversive stimuli.^40^ Aversive images of threat or injury were contrasted with neutral images of human or inanimate objects selected from the International Affective Picture System (IAPS: https://csea.phhp.ufl.edu/media/iapsmessage.html). Any images with alcohol or drugs in them were not included. Similar tasks have been used to demonstrate functional activation in alcohol- and drug-dependent groups in response to negative IAPS images.^38, 39^ A total of 240 images (120 aversive, 120 neutral) were presented in a block design consisting of two runs. Each run contained eight blocks of six images presented for 5 s each, followed by a 400 ms inter-stimulus interval that consisted of a fixation cross. Participants were given the implicit task to press a response button when the next image appeared on the screen. Images were counterbalanced for valence and arousal between sessions and between blocks and were presented in a pseudorandomized order, with a neutral image always presented first. Each block was separated by a 15 s rest period to prevent habituation effects. Response latency (in ms) was recorded for each participant in response to all images presented.

### Statistical analysis

#### Demographic, baseline assessment and behavioral measures

Demographic and baseline assessment measures were analyzed using univariate analysis of variance (ANOVAs) for continuous variables and chi-square for categorical variables. *Post-hoc* Tukey tests were used to identify pairwise differences for continuous variables. Mean response latencies during emotional processing were square-root transformed to stabilize variances relative to the mean (untransformed scores presented in Table 1). Repeated-measures ANOVAs were used with the two within-subject factors condition (neutral, aversive) and treatment (placebo, naltrexone) and the between-subject factor group (control, alcohol, alcohol/drugs) to examine response latencies.

Repeated-measures ANOVAs were also used to evaluate changes in affective states between the experimental medicine visits. To determine the influence of naltrexone on task performance, we calculated a change score for each individual by subtracting mean latencies from each condition (aversive minus neutral) following placebo and naltrexone. We then used Pearson's *r* coefficients to test for associations between change scores and individual variations in affective states.

#### Neuroimaging data acquisition, processing and analysis

Volumetric MRI data was acquired with 3 T systems at three sites (Imanova, London; Wolfson Brain Imaging Centre, Cambridge; and the Translational Imaging Unit, Manchester). London and Cambridge operated nominally identical 3 T Siemens Tim Trio systems and Manchester operated a 3 T Philips Achieva.

fMRI data were preprocessed using SPM8. Mean echo-planar imaging (EPI) was first computed for each participant and visually inspected in the orbitofrontal cortex and temporal lobe to ensure that none showed excessive signal dropout. All EPIs were then realigned to the first scan by rigid body transformations to correct for head movements. Next, EPIs were normalized to the standard template in the Montreal Neurological Institute (MNI) space using linear and nonlinear transformations and smoothed with a Gaussian kernel of full width at half maximum of 8 mm. Realignment parameters were then inspected for each subject to make sure that movements of translation and rotation were <2 mm and 2°, respectively.

To identify significant differences across groups in regional responses of brain areas, a general linear model (GLM) was employed. Subject-specific GLMs included three experimental factors (aversive images, neutral images and fixation cross) and six realignment parameters as effects of no interest to account for residual motion-related variance. Low-frequency signal drift was removed using a high-pass filter (cut-off 128 s). An autoregressive modeling of temporal autocorrelations was applied. The following contrasts were generated for the second-level analysis: (1) aversive images versus neutral images, (2) aversive images versus fixation cross and (3) neutral images versus fixation cross.

Group differences in brain responses were assessed via: (1) a full factorial analysis for the contrast ‘aversive versus neutral' including three groups (control, alcohol, alcohol/drugs) and two pharmacological treatments (placebo, naltrexone) as main factors and (2) a full factorial analysis including three groups (control, alcohol, alcohol/drugs), two pharmacological treatments (placebo, naltrexone) and two task conditions (aversive images versus fixation cross, neutral images versus fixation cross) as main factors. For each model, SPM-F-maps assessing the main effect of group, the main effect of treatment, the group-by-treatment interaction and the group-by-treatment-by-condition interaction were generated, respectively. An additional covariate of site was added to remove the effect of the different scan sites. In the first model, we also included the abuse composite score as a covariate-of-interest to test for associations between brain response and childhood adversity as a function of pharmacological treatment.

To threshold the second-level maps, we used *a priori* regions of interest (ROIs) based on a functional definition of the brain regions as previously recommended (*P*<0.05, family-wise error (FWE) correction for multiple comparisons after a small volume correction).^56, 57, 58, 59^ This commonly employed statistical procedure not only ensures a robust protection against type I errors but also prevents false negative results.

Here, the following anatomical ROIs were selected because of their functional role in negative emotional processing in substance use disorders:^1, 10, 11, 12, 13, 14, 15, 16, 38, 39^ the amygdala, the mPFC and the hippocampus. Each ROI was defined using a sphere centered on MNI coordinates obtained from previous studies.^60, 61^ Specifically, a sphere with a radius of 5 mm was centered on the right amygdala (*x*,*y*,*z*: 25,−3,−27), a sphere with a radius of 10 mm was centered on the right mPFC (*x*,*y*,*z*: 14,62,1) and a sphere with a radius of 8 mm was centered on the right hippocampus (*x*,*y*,*z*: 29,−14,−14). The different size of the spheres was chosen to reflect the actual size of the regions. Brain regions that were not predicted *a priori* but met a threshold of *P*<0.05, whole-brain corrected, were also reported.

#### Functional connectivity: psycho-physiological interaction analyses

Psycho-physiological interaction (PPI) represents the change in connectivity between a seed region and the rest of the brain that is induced by a specific psychological context. The ACC was chosen as a seed due to the high density of MORs in this region that potentially reflects higher binding potential associated with greater capacity to modulate negative emotional processing.^62^ We sought to identify brain regions that had a differential functional connectivity pattern with the seed region during the processing of aversive versus neutral images. For each participant, a 15 mm sphere was centered on the right anterior cingulate cortex (*x*,*y*,*z*: 6,60,4; extracted from the first model). The time series of the BOLD response for each participant was then computed using the first eigenvariate from all voxels' time series in the sphere.

The BOLD time series for each individual was deconvolved to estimate a neuronal time series for the source, using the PPIs deconvolution parameter defaults in SPM8.^63^ The PPI regressor was calculated as the element-by-element product of the seed neuronal time series and a vector coding for the main effect of task (1 for aversive images, −1 for neutral images). This product was reconvolved by the canonical haemodynamic response function. The statistical model also included the main effect of the task convolved by the haemodynamic response function, the seed neuronal time series and the six movement parameters as effects of no interest.

Subject-specific PPI models were run, and contrast images were generated such that the identified target regions were those that showed a change in connectivity with the ACC during the processing of aversive versus neutral images. Subject-specific PPI contrast images were entered into second-level GLMs to assess if change in connectivity between the ACC and other regions in the brain were associated with childhood adversity as a function of pharmacological treatment. The same statistical approaches previously described were employed to threshold the second-level PPI maps.

## Results

### Demographic information and baseline assessment

As shown in Table 2, the three groups did not differ with regard to gender, age, verbal intelligence, handedness, smoking status and locus of control beliefs (all *P*'s>0.05). However, significant group differences emerged with regard to stress sensitivity (F_2,57_=5.71, *P*=0.005) and childhood adversity (F_2,57_=6.63, *P*=0.003), such that the alcohol/drugs group reported significantly higher sensitivity to stress compared with the control group (Tukey's *P*=0.004) and significantly higher levels of childhood adversity compared with the control (Tukey's *P*=0.005) and alcohol (Tukey's *P*=0.012) groups.

The groups also differed with regard to affective states (BDI-II, STAI-State) on the day of testing, as assessed prior to scanning on placebo (F_2,57_=6.37, *P*=0.003; F_2,57_=8.42, *P*=0.001) and naltrexone (F_2,57_=5.04, *P*=0.010; F_2,57_=3.93, *P*=0.025). The alcohol/drugs group scored significantly higher than the control group on total scores for both measures (both Tukey's *P*=0.002). However, affective state did not significantly fluctuate between the two experimental medicine sessions, as reflected by non-significant group-by-session interactions (BDI-II: F_2,57_=.64, *P*=0.534; STAI-State: F_2,57_=2.13, *P*=0.129), and the main effects of treatment were not significant for either measure (BDI-II: F_1,57_=1.41, *P*=0.240; STAI-State: F_1,57_=1.72, *P*=0.194). Total scores of the BDI-II and STAI-State did not correlate significantly with behavioral change scores (all *P*'s>0.05) and consequently were not used as covariates in subsequent behavioral analyses. As we did not identify differences in demographics between participants recruited at the three different sites (all *P*'s>0.2), we did not include scanning site as a covariate in behavioral analyses.

### Task-related performance during evocative image processing

As shown in Table 1, participants in both the alcohol and alcohol/drugs groups tended to respond more slowly in general, but there was considerable variability and differences between the groups failed to reach significance (F_2,57_=3.04, *P*=0.056). There was a main effect of condition (aversive versus neutral images; F_1,57_=11.27, *P*=0.001), such that response latency to aversive images (818.87 ms s.d. ±343.55) was significantly longer than response latency to neutral images (758.11 ms ±271.47) (*t*_59_=−3.36, *P*=0.001) across groups. However, there was no main effect of treatment (F_1,57_=0.006, *P*=0.941) and the group-by-treatment (F_2,57_=0.90, *P*=0.915) and the group-by-treatment-by-condition (F_2,57_=0.45, *P*=0.640) interactions were also not significant.

### Task-related brain activation during evocative image processing

**Figure 1:**
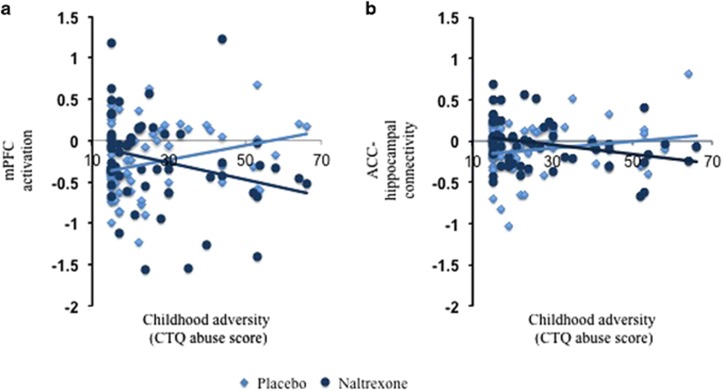
Naltrexone modulated (**a**) task-related activation in the right medial prefrontal cortex (mPFC; MNI coordinates: *x*,*y*,*z*: 20,60,0; *z*-score=3.71, *P*=0.004) and (**b**) functional connectivity between the right anterior cingulate cortex (ACC; seed coordinates: *x*,*y*,*z*: −14,62,1) and the right hippocampus (MNI coordinates: *x*,*y*,*z*: 22,−16, −10; *z*-score=3.11; *P*=0.002) as a function of childhood adversity (Childhood Trauma Questionnaire (CTQ) abuse score) in all groups.

Significant correlations between the ROIs and childhood adversity were found during the processing of aversive versus neutral images as a function of pharmacological treatment. Figure 1a shows a positive association between activity in the right mPFC (*x*,*y*,*z*: 20,60,0) and childhood adversity (*z*-score=3.71, *P*=0.004 FWE for multiple comparisons after small volume correction) and, similarly, Figure 1b shows a positive association between functional connectivity between the right ACC and the right hippocampus (*x*,*y*,*z*: 22,−16,−10) and childhood adversity (*z*-score=3.11; *P*=0.002, FWE for multiple comparisons after small volume correction) in the placebo relative to the naltrexone session. This pattern of association was found irrespective of group.

**Figure 2:**
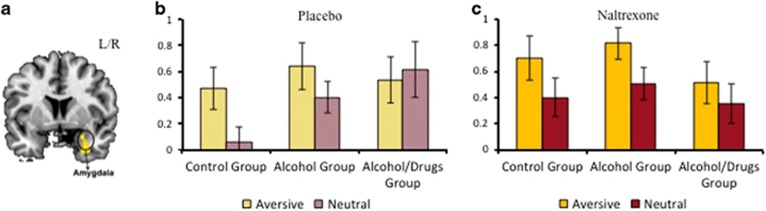
(**a**) Significant group-by-treatment-by-condition interaction in the right amygdala (MNI coordinates: *x*,*y*,*z*: 26,2,−24; *z*-score=2.66; *P*=0.032). (**b**) On placebo, the control and alcohol groups showed increased activation to aversive relative to neutral images, whereas the alcohol/drugs groups showed increased activation to all visual images, irrespective of emotional valence. (**c**) Naltrexone normalized activation in the alcohol/drugs group, such that activation was higher to aversive relative to neutral images, but did not change the pattern of activation in the control or alcohol groups. MNI, Montreal Neurological Institute.

As shown in Table 3, there was a main effect of group and a main effect of condition in temporal–occipital regions. There was a significant group-by-treatment-by-condition interaction (*z*-score=2.66; *P*=0.032 FWE for multiple comparisons after small volume correction) in the right amygdala (*x*,*y*,*z*: 26,2,−24; Figure 2) while viewing aversive and neutral images relative to baseline. Figure 2a shows that the alcohol and control groups had higher activation in the amygdala when processing aversive relative to neutral images on placebo, thus demonstrating increased activation to threat-related stimuli. However, activation in the amygdala increased to *both* aversive and neutral images in the alcohol/drugs group, irrespective of emotional valence.

Figure 2b shows that the group-by-treatment-by-condition interaction was mainly driven by a normalization of response in the amygdala for aversive relative to neutral images under naltrexone in the alcohol/drugs group, whereas the pattern of activation did not change in the alcohol and control groups. There was no main effect of treatment and the group-by-treatment interaction was not significant.

## Discussion

### Influence of childhood adversity on naltrexone

Childhood adversity was highly prevalent in our alcohol/drugs group and significantly differed between the patient groups. This is in line with previous studies showing that individuals with a history of childhood abuse are more prone to combined alcohol and drug taking in adulthood.^31, 32, 33^ Sensitivity to stress was also high in our alcohol/drugs group compared with the control group. These differences support that aversive emotional states contribute to negatively reinforced drug seeking, consistent with the ‘dark side' view of addiction.^8, 9^ Negative reinforcement thus acts to increase more compulsive drug-seeking behavior, particularly during the withdrawal/negative affect stage of the addiction cycle.^64^

Similarities between the effects of childhood maltreatment, one form of early adversity, and impairments in emotion regulation systems underlying vulnerability to substance use disorders have been identified, but are poorly understood.^65^ In maltreated adolescents, increased activation in prefrontal regions has been found during effortful control of emotion regulation.^66^ However, surprisingly few fMRI studies have investigated brain response to emotional stimuli in substance-dependent groups abused in childhood. It has been shown that functional alterations in mPFC activity are associated with greater maltreatment severity in response to drug- and stress-related cues in individuals with cocaine dependence.^67^ Heighted mesolimbic response has also been found in response to drug-related and evocative cues in cocaine-dependent individuals reporting a history of emotional, physical and sexual abuse.^68^ Consistent with preclinical evidence demonstrating that the therapeutic efficacy of naltrexone is associated with early adverse experiences,^29^ we found that naltrexone modulated task-related activation in the mPFC, one key region associated with emotion dysregulation in both substance use disorders and adults with histories of childhood adversity, depending on the degree of abuse experienced.

Naltrexone also modulated ACC–hippocampal connectivity, two regions integral to mood regulation,^69^ depending on childhood adversity. In fMRI studies, hippocampal activation and network connectivity has been shown to predict cocaine relapse.^12^ Limbic connectivity between the hippocampus and the amygdala is also associated with reduced adaptive emotional processing in maltreated individuals with methamphetamine dependence.^11^ Interplay between the ACC and the hippocampus has been implicated in the reconsolidation and expression of fear memories,^70^ with alterations in ACC–hippocampal connectivity found in patients with posttraumatic stress disorder.^71^ These findings, together with our data, suggests that opioid receptors in the ACC and the hippocampus are important pharmacological targets in individuals with a history of abuse, which, although not specific to our substance-dependent groups, may be of relevance to other disorders characterized by childhood adversity, such as trauma-related or psychotic disorders.

The neurobiological effects of childhood adversity on emotional processing are under-investigated in addiction. Our data confirm that environmental factors, specifically early adverse experiences, influence variations in naltrexone response. It is possible that differences in childhood adversity might account for some of the variability in outcome in alcohol-dependent patients treated with naltrexone. Early life experiences have been shown to affect the endogenous opioid system in preclinical models,^72, 73^ and the present data offer convergent evidence that the effects of childhood adversity on endogenous opioids are an important indicator of responsiveness to pharmacological treatments acting on MOR transmission in humans. We suggest that behavioral interventions that promote emotion regulation strategies for traumatic experiences or pharmacological interventions that normalize cortical–limbic connectivity may be effective in groups with high levels of childhood adversity.

### Effects of MOR blockade on different types of substance dependence

Previous fMRI studies have shown altered limbic activation in response to naturally evocative,^16^ negative emotional^37, 38^ and substance-related^74, 75, 76, 77, 78^ stimuli in substance-dependent groups. We observed greater task-related activation in the right amygdala during the processing of aversive relative to neutral images under placebo in individuals recovering from alcohol dependence. Heightened reactivity in this region is consistent with previous investigations measuring brain response to negative IAPS images in individuals with alcohol dependence.^38^ Similar to our alcohol group, participants from the above studies were characterized by only one drug dependency. The lack of discriminatory response between aversive and neutral images was specific to our alcohol/drugs group, possibly reflecting an emotional desensitization following prior exposure to adverse events, an attentional disengagement to aversive images (although we consider this unlikely as our task does not tap into attentional resources) or altered MOR availability/density associated with abstinence from repeated drug exposure (for example, cocaine;^79, 80^ opiates;^81^ alcohol^82, 83^) and/or individual differences in environment factors. Although speculative for the present study, these possibilities each deserve more detailed investigation in substance-dependent groups, including use of positron emission tomography to elucidate whether associations between MOR binding potential and limbic activation differ during negative emotional processing between alcohol-dependent patients with and without combined drug dependence.

Our data further showed that naltrexone normalized the pattern of activation in the amygdala for aversive relative to neutral images in our alcohol/drugs group (that is, there was only an effect of treatment at the group level when interacting with the content of the images presented). These data demonstrate that naltrexone-induced functional alterations during negative emotional processing are specific to groups characterized by distinct patterns of drug dependencies that can persist in prolonged abstinence. Activation of stress-induced neurocircuitry contributes to the ‘dark side' view of addiction, in which recruitment of brain ‘anti-reward' systems (for example, within motivational circuits of the extended amygdala), an opponent process to hyperactivation of the brain reward system, provides strong motivation for negatively reinforced drug seeking.^64, 84^ Increased activation in response to aversive images could also lend vulnerability to craving and relapse via stress-induced reinstatement of emotion-processing circuitry.^8, 9, 84^ However, a relative normalization of response by naltrexone in our alcohol/drugs group is likely due to a kappa-antagonist effect being more potent in individuals with increased dynorphin-dependent KOR activation. The latter is associated with negative emotional states, higher sensitivity to stress and/or more severe psychopathology as a consequence of combined drug taking.^85^ Stress-induced activation of the dynorphin/kappa-opioid system has dysphoric-like effects that are thought to mediate negative emotional states.^86, 87^ However, KOR blockade has shown consistent anxiolytic and antidepressant effects in both humans and animals, including attenuation of a stress response.^88, 89^ Evaluation of the potential therapeutic benefits of selective kappa-opioid antagonism in the treatment of addiction and comorbid stress-related mood disorders thus may form an important area of new research. Increasing activation of the HPA axis with mu-opioid antagonism (including adrenocorticotropic hormone, beta-endorphin and cortisol levels) is also a potential mechanism for reducing craving and withdrawal symptoms in alcohol addiction.

### Treatment implications

Naltrexone significantly reduces return to heavy drinking (to 83% of the risk of placebo) and decreases drinking days by about 4%.^90^ In individuals with opioid dependence, the efficacy of naltrexone treatment is largely heterogeneous and most effective in subgroups that complete treatment.^91^ However, medication compliance and retention rates remain poor (28%), particularly during early stages of recovery.^92^ This is likely explained by unwanted side effects such as sedation (for example, daytime sleepiness) and gastrointestinal problems (for example, nausea, stomach pain, loss of appetite).^90^ Extended-release naltrexone has thus been developed and used, with success, to improve adherence over and above other pharmacotherapies used in the treatment of alcohol and opioid dependence (for example, acamprosate, buprenorphine).^93^

Encouragingly, cognitive behavioral therapy with adjunct naltrexone treatment has shown to reduce return to drinking in alcohol-dependent adults, thus suggesting beneficial, synergistic effects of concurrent therapies.^94^ Cognitive behavioral therapy also has the potential to improve functional abnormalities during emotional processing (in the medial prefrontal and anterior cingulate cortices, for example), as shown in patients with depression.^95, 96^ Interventions that combine naltrexone and coping skills have shown to improve treatment outcome in alcohol and cocaine dependence.^97, 98, 99^ Coping skills therapies with a particular focus on relapse prevention may also help naltrexone-treated patients better manage their emotions after non-substance using strategies are implemented. As the current treatment length of naltrexone is recommended for 6 months,^17^ coping skills for childhood adversity should be addressed and evaluated as part of a longer-term therapeutic process for addiction recovery.

### Limitations

The main limitation of our analyses is its use of a retrospective self-report measure of childhood adversity. Measurement of an emotional or traumatic event may be unreliable as recollection is likely to be influenced by several confounding factors including memory biases, memory repression and current mood; however, retrospective recall of physical and sexual abuse is associated with more false negatives than false positives,^100^ suggesting a greater tendency for adults to under- rather than to overestimate the occurrence of an abusive experience.

Secondly, our study only investigated the effects of naltrexone on the processing of aversive images compared with neutral images. Comparison with positive or rewarding non-alcohol and -drug-related images would have allowed us to investigate if the valence of emotional processing had a differential treatment effect, or if motivational salience had the same effect irrespective of emotionality.

Lastly, childhood adversity is a robust risk factor for other disorders in which emotional problems pervade. Major depressive disorder, for example, is associated with abnormal responses to negative emotional stimuli in similar brain regions observed here.^95, 96^ It is possible that variations in anxiety and depression could also drive abnormalities in negative emotional processing in substance use disorders, or that more severe comorbidity has a particularly potent effect for enhancing emotion-processing circuitry. Although we cannot preclude this possibility from the current data, levels of anxiety and depression were consistent in groups across the experimental medicine sessions (and therefore exerting the same influence on emotional processing at both time points), and sensitivity to stress did not differ between the patient groups at baseline.

## Conclusions

Problems with emotion regulation may predate substance dependence, or may serve to exacerbate susceptibility to relapse during addiction recovery. Furthermore, loss of brain reward and recruitment of brain stress systems produces negatively reinforced motivation for compulsive drug seeking and addiction. The present study demonstrates that the effects of naltrexone on cortical activation and cortical–limbic connectivity are dependent on variations in childhood adversity (that is, the more abuse experienced, the greater the ameliorative effect of naltrexone). Pharmacotherapy with naltrexone may also have some ameliorative effects on negative emotional processing in the amygdala in individuals with combined alcohol and drug dependence, but not alcohol dependence alone, possibly because of alterations in endogenous opioid transmission or the KOR antagonist actions of naltrexone. Childhood adversity was higher in individuals with combined alcohol and drug dependence compared with individuals dependent on alcohol only and should thus be an important consideration by treatment strategies for addiction. We conclude that childhood adversity is one environmental factor that influences pharmacological response to naltrexone. More tailored treatment approaches are required that take into account such early experiential factors.

## Figures and Tables

**Table 1:** Evocative Images task mean group latencies (ms) and standard deviations in response to neutral and aversive images on placebo and naltrexone

*Condition*	*Treatment*	*Control group,* n=*21*	*Alcohol group*, n=*18*	*Alcohol/drugs group,* n=*21*
Neutral images	Placebo	691.14 (±294.94)	862.17 (±291.95)	740.62 (±313.31)
Neutral images	Naltrexone	671.14 (±237)	893.50 (±354.28)	724.29 (±219.06)
Aversive images	Placebo	684.48 (±261.16)	945.94 (±368.01)	855.71 (±457.75)
Aversive images	Naltrexone	689.76 (±238.30)	970.39 (±478.30)	806.71 (±382.78)

Means are untransformed scores.

**Table 2:** Group demographic information and baseline assessment prior to the experimental medicine sessions

	*Control group*, n=*21*	*Alcohol group*, n=*18*	*Alcohol/drugs group*, n=*21*	*Statistic,* P*-value*
Site (London: Cambridge: Manchester)	8 L: 12 C: 1 M	9 L: 6 C: 3M	12 L: 7 C: 2 M	*X*^2^=4.04, *P*=0.40
Gender (male: female)	17 M: 4 F	14 M: 4 F	16 M: 5 F	*X*^2^=0.15, *P*=0.93
Age (years)	41.52 (±10.05)	44.22 (±8.72)	40.57 (±7.43)	F_2,57_=0.88, *P*=0.42
Verbal IQ (WTAR)	106.57 (±10.88)	105.78 (±8.31)	99.57 (±11.45)	F_2,57_=2.80, *P*=0.07
Handedness (Edinburgh Inventory)	40.62 (±67.71)	58.39 (±66.72)	61.33 (±58.06)	F_2,57_=0.63, *P*=0.54
Smoking (smoker: non-smoker)	9 S: 12 N-S	13 S: 5 N-S	16 S: 5 N-S	*X*^2^=5.90, *P*=0.052
Stress sensitivity (PSS-14, total score)	15.10 (±6.36)	18 (±7.23)	21.95 (±6.27)	F_2,57_=5.71, *P*=0.005
Childhood adversity (CTQ total abuse score)	21.90 (±10.07)	22.61 (±12.06)	34.24 (±16.50)	F_2,57_=6.63, *P*=0.003
Locus of control (Rotter's I–E)	10.25 (±43.18)	10.76 (±43.39)	10.40 (±43.28)	F_2,57_=0.05, *P*=0.95
Drug-related locus of control (DR-LOC recovery)	1.24 (±1.61)	1.50 (±1.58)	1.48 (±.98)	F_2,57_=0.21, *P*=0.81

Abbreviations: CTQ, Childhood Trauma Questionnaire; DR-LOC, drug-related locus of control; PSS-14, Perceived Stress Scale; Rotter's I–E, Rotter's internal–external control scale; WTAR, Wechsler Test of Adult Reading.

**Table 3:** Significant whole-brain-corrected regions for the main effect of group, main effect of condition and group-by-treatment-by-condition interaction

	*Hemisphere*	P*-value*	*z-score*	*Coordinates (x:y:z)*
*Main effect of group*				
Fusiform gyrus	Left	0.012	4.83	−26, −66, −16
Inferior occipital gyrus	Left	0.025	4.66	−26, −82, −6
Precuneus	Right	0.029	4.63	30, −60, 28
				
*Main effect of condition*				
Inferior temporal gyrus	Right	0.001	5.27	46, −44, −18
Superior temporal gyrus	Left	0.023	4.66	−58, −12, 4
Middle occipital gyrus	Left	0.028	4.61	−44, −80, −2
				
*Group-by-treatment-by-condition interaction*				
** **Amygdala	Right	0.032	2.66	26, 2, −24
